# The impact of measurement errors in the identification of regulatory networks

**DOI:** 10.1186/1471-2105-10-412

**Published:** 2009-12-13

**Authors:** André Fujita, Alexandre G Patriota, João R Sato, Satoru Miyano

**Affiliations:** 1 Computational Science Research Program, RIKEN, 2-1 Hirosawa, Wako, Saitama, 351-0198, Japan; 2 Institute of Mathematics and Statistics, University of São Paulo, Rua do Matão, 1010 - São Paulo, 05508-090, Brazil; 3 Center of Mathematics, Computation and Cognition, Universidade Federal do ABC, Rua Santa Adélia, 166 - Santo André, 09210-170, Brazil; 4 Human Genome Center, Institute of Medical Science, University of Tokyo, 4-6-1 Shirokanedai, Minato-ku, Tokyo, 108-8639, Japan

## Abstract

**Background:**

There are several studies in the literature depicting measurement error in gene expression data and also, several others about regulatory network models. However, only a little fraction describes a combination of measurement error in mathematical regulatory networks and shows how to identify these networks under different rates of noise.

**Results:**

This article investigates the effects of measurement error on the estimation of the parameters in regulatory networks. Simulation studies indicate that, in both time series (dependent) and non-time series (independent) data, the measurement error strongly affects the estimated parameters of the regulatory network models, biasing them as predicted by the theory. Moreover, when testing the parameters of the regulatory network models, p-values computed by ignoring the measurement error are not reliable, since the rate of false positives are not controlled under the null hypothesis. In order to overcome these problems, we present an improved version of the Ordinary Least Square estimator in independent (regression models) and dependent (autoregressive models) data when the variables are subject to noises. Moreover, measurement error estimation procedures for microarrays are also described. Simulation results also show that both corrected methods perform better than the standard ones (i.e., ignoring measurement error). The proposed methodologies are illustrated using microarray data from lung cancer patients and mouse liver time series data.

**Conclusions:**

Measurement error dangerously affects the identification of regulatory network models, thus, they must be reduced or taken into account in order to avoid erroneous conclusions. This could be one of the reasons for high biological false positive rates identified in actual regulatory network models.

## Background

There has been an increasing interest among bioinformaticians in the problem of quantifying correctly gene expression in a given sample. It is well accepted that the observed gene expression value is a combination of the "true" gene expression signal with intrinsic biological variation (natural fluctuation) and a variation caused by the measuring process, also known as measurement error. Studies have documented the presence of sizable measurement error in data collected mainly from microarrays and also by other approaches such as Real Time RT-PCR, Northern blot, CAGE, SAGE, etc [1,2]. This measurement error can be easily observed when two technical replicates are plotted in a MA (M is the logarithm of the intensity ratio and A is the mean of the logged intensities for a dot in the plot) or scatter plots. Frequently, a considerable dispersion can be observed. This dispersion is due to the measurement error, since, in theory, technical replicates (same samples) must present the same quantifications. In general, these fluctuations are derived from probe sequence, hybridization problems, high background fluorescence, signal quantification procedures (image analysis), etc [3,4]. In the last few years, a considerable number of reports on the problem of quantifying and separating "true" gene expression signal from noise [5-7] has been published with the main aim to find differentially expressed genes [8,9]. Despite these results in gene expression analysis and a large amount of research performed in modeling regulatory networks (Bayesian networks [10,11], Boolean networks [12,13], Relevance networks [14], Graphical Gaussian models [15], Differential equations [16], etc), only a fraction of the statistical studies use procedures designed for modeling networks taking into account measurement error.

Frequently, Ordinary Least Squares (OLS) and methods related to it, such as Pearson and Spearman correlations [17], ridge, lasso and elastic net regressions [18] are widely used as estimators to quantify the strength of association between gene expression signals and model regulatory network structures. In the time series context, estimation process of Autoregressive (AR) [19-22] models also use OLS to identify which gene is or is not Granger causing another gene. Generally, a regression is carried out between the target gene and its potential predictors in order to test which predictor gene has, at a gene expression level, association with the target gene.

It is well known in the statistical literature that, when the measurement errors are ignored in the estimation process, OLS and its variants become inconsistent (i.e., even increasing the sample size the estimates do not converge to the true values). More precisely, the estimation of the slope parameters is attenuated [23] and consequently, regulatory network models become biased. Moreover, there is no control of type I error since standard OLS was not designed to treat measurement error. In this context, an adequate inference treatment must be considered for the model parameters in order to avoid inconsistent estimators. Usually, measurement equations are added to the model to capture the measurement errors effect, therefore, producing consistent estimators, efficient and asymptotically normally distributed. A careful and deep exposition on the inferential process is presented in [23] and the references therein. Although there are studies referring to problems caused by measurement errors in the statistical literature, there is a gap in the network modeling theory which must be filled in to avoid misinterpretation and distort conclusions from the inferential process. Here, we focus on the development and present some important statistical tools to be applied in OLS-based and VAR network models taking into account the measurement errors effect. We also conduct simulation studies in order to evaluate the impact of the measurement error in the identification of gene regulatory networks using the standard OLS in both conditions, time series and non time series data. Surprisingly, both the simulations and theory described that, in the presence of measurement error, the estimated coefficients are biased even increasing the amount of observations, and the statistical tests are not controlling the rate of false positives properly. These results were also observed in time series context, where the autoregressive coefficients were strongly affected. Thus, a corrected version of the OLS estimator for independent (in the regression context) and dependent (in the autoregressive context) data containing measurement error were developed. Results in both, simulated and actual biological data are illustrated. Moreover, two procedures to estimate measurement error in microarrays are presented.

## Results and discussions

In order to evaluate the performance of conventional OLS and VAR methods in practice, simulations were carried out in artificial data with absence and presence of measurement error. Noise was added at different rates, and sample size was increased in order to evaluate the consistence of conventional and proposed approaches.

In the following we give a brief explanation about the usual and proposed methods. Let *x *and *y *be variables (gene expression values) with the following relationship *y *= *α *+ *βx *+ *ε*, where *ε *is the random error (intrinsic biological variation) of the model with zero mean and finite variance. In general, we are interested in estimating the parameters *α *and *β *to make inferences about them. In practice, we take a sample *x*_*i*_, *y*_*i *_for *i *= 1,..., *n *and use these quantities to obtain estimates for the parameters of interest. However, it is not always possible to observe directly the values of *x *and *y *because sometimes they are latent values, i.e., they are masked by measurement errors derived by the measurement process in microarrays, for example. Then, instead of observing the true variables, we observe surrogate variables *X *and *Y *which carry an error, that is *X *= *x *+ ϵ_1 _and *Y *= *y *+ ϵ_2_, where ϵ_1 _and ϵ_2 _are measurement errors. Generally, what is done in practice is a naive solution, since it simply replaces *x *with *X *and *y *with *Y *in the regression equation and uses the OLS approach to estimate the parameters. That is, based on the equation *Y *= *α *+ *βX *+ *ε*, estimators are built. On the other hand, the proposed approach is slightly different. The latter considers three equations, namely: *y *= *α *+ *βx *+ *ε*, *X *= *x *+ ϵ_1 _and *Y *= *y *+ ϵ_2 _and uses them to estimate the model parameters. This little difference can result great impact in the estimators properties of each approach. Notice that the former produces inconsistent estimators and the latter produces consistent estimators when the data contains measurement error. The same idea can be applied in the time series context.

The corrected versions of the OLS estimators in both independent and dependent data were compared to their conventional forms in order to evaluate the performance under gene expression data containing measurement error. The standard OLS and VAR models are particular cases of the proposed models in the case when the measurement error is absent. Firstly, simulations were performed in regression models. Table 1 illustrates average coefficients estimated by standard OLS in 10,000 Monte Carlo simulations. Notice that increasing the rate of measurement error, more attenuated become the estimated coefficients, i.e., the estimates are shifted towards zero. Table 2 illustrates the percentage of rejected hypotheses in 10,000 Monte Carlo simulations. Analyzing when *β*_1 _= 0, i.e., when there is no association between the corresponding covariate and the response variable, Table 2 shows that the OLS approach does not control, at a 5% nominal level, the rate of false positives. The larger the sample size, the worst the OLS performance, as it was expected to be. On the other hand, the coefficients of the corrected OLS are unbiased (Table 1 - values between brackets) and converge to "true" value when sample's size becomes larger. Moreover, the rate of false positives are actually controlled under the null hypothesis (Table 2 - values between brackets).

**Table 1:** Ordinary least squares.

EM	n	***β***_**1**_	***β***_**2**_	***β***_**3**_	***β***_**4**_	***β***_**5**_	***β***_**6**_	***β***_**7**_	***β***_**8**_	***β***_**9**_
		0	-0.1	-0.2	-0.3	-0.4	0.5	0.6	0.7	0.8
										
0	50	0.00	-0.10	-0.20	-0.30	-0.40	0.50	0.60	0.70	0.80
	100	0.00	-0.10	-0.20	-0.30	-0.40	0.50	0.60	0.70	0.80
	200	0.00	-0.10	-0.20	-0.30	-0.40	0.50	0.60	0.70	0.80
	400	0.00	-0.10	-0.20	-0.30	-0.40	0.50	0.60	0.70	0.80
										
0.2	50	0.01 (0.00)	-0.09 (-0.11)	-0.18 (-0.20)	-0.28 (-0.30)	-0.37 (-0.41)	0.48 (0.50)	0.58 (0.61)	0.67 (0.71)	0.76 (0.81)
	100	0.01 (0.00)	-0.09 (-0.10)	-0.18 (-0.20)	-0.28 (-0.30)	-0.38 (-0.40)	0.48 (0.50)	0.58 (0.60)	0.67 (0.70)	0.77 (0.80)
	200	0.01 (0.00)	-0.09 (-0.10)	-0.19 (-0.20)	-0.28 (-0.30)	-0.38 (-0.40)	0.48 (0.50)	0.58 (0.60)	0.67 (0.70)	0.77 (0.80)
	400	0.01 (0.00)	-0.09 (-0.10)	-0.18 (-0.20)	-0.28 (-0.30)	-0.37 (-0.40)	0.48 (0.50)	0.58 (0.60)	0.67 (0.70)	0.77 (0.80)
										
0.4	50	-	-	-	-	-	-	-	-	-
	100	0.02 (0.00)	-0.07 (-0.11)	-0.15 (-0.21)	-0.23 (-0.31)	-0.31 (-0.42)	0.44 (0.51)	0.52 (0.62)	0.60 (0.72)	0.69 (0.82)
	200	0.02 (0.00)	-0.06 (-0.10)	-0.15 (-0.20)	-0.23 (-0.31)	-0.31 (-0.40)	0.44 (0.51)	0.52 (0.61)	0.60 (0.71)	0.69 (0.81)
	400	0.02 (0.00)	-0.06 (-0.10)	-0.15 (-0.20)	-0.23 (-0.30)	-0.31 (-0.40)	0.44 (0.50)	0.52 (0.60)	0.60 (0.70)	0.69 (0.80)
										
0.6	50	-	-	-	-	-	-	-	-	-
	100	-	-	-	-	-	-	-	-	-
	200	0.03 (-0.01)	-0.04 (-0.11)	-0.10 (-0.21)	-0.17 (-0.32)	-0.24 (-0.42)	0.38 (0.52)	0.45 (0.62)	0.52 (0.72)	0.58 (0.82)
	400	0.03 (0.00)	-0.04 (-0.10)	-0.10 (-0.20)	-0.17 (-0.31)	-0.24 (-0.41)	0.38 (0.51)	0.45 (0.61)	0.52 (0.71)	0.58 (0.81)
										
0.8	50	-	-	-	-	-	-	-	-	-
	100	-	-	-	-	-	-	-	-	-
	200	-	-	-	-	-	-	-	-	-
	400	0.05 (0.00)	-0.01 (-0.11)	-0.07 (-0.21)	-0.12 (-0.32)	-0.18 (-0.42)	0.32 (0.51)	0.38 (0.62)	0.43 (0.72)	0.49 (0.83)

Average OLS estimated coefficients and corrected OLS (between brackets) in 10,000 simulations. The model is described in (Simulations section, simulation I - independent data). "-" means that it was not possible to calculate due to high measurement error in comparison to sample's size. EM: Standard deviation of the Error of Measure. n: Number of samples. Notice that, in the presence of measurement error, the coefficients (*β*) estimated by the corrected OLS (between brackets) converge to the "true" values, while the estimated by standard OLS do not.

**Table 2:** Ordinary least squares.

EM	n	***β***_**1**_	***β***_**2**_	***β***_**3**_	***β***_**4**_	***β***_**5**_	***β***_**6**_	***β***_**7**_	***β***_**8**_	***β***_**9**_
		0	-0.1	-0.2	-0.3	-0.4	0.5	0.6	0.7	0.8
										
0	50	**4.94**	9.06	21.57	41.73	63.19	81.09	92.15	97.24	99.03
	100	**4.90**	13.82	42.31	74.11	93.20	98.83	99.87	100.0	100.0
	200	**4.99**	25.62	72.24	96.64	99.95	99.99	100.0	100.0	100.0
	400	**5.17**	44.31	95.53	99.98	100.0	100.0	100.0	100.0	100.0
										
0.2	50	**4.81 **(**4.73**)	8.24 (8.45)	17.52 (18.12)	34.39 (34.94)	54.86 (55.38)	76.09 (73.71)	88.70 (86.95)	95.40 (94.61)	98.12 (97.69)
	100	**5.30 **(**5.20**)	11.35 (12.27)	34.69 (36.27)	65.53 (67.16)	88.92 (89.57)	98.22 (97.81)	99.76 (99.66)	99.97 (99.96)	100.0 (100.0)
	200	**5.23 **(**5.25**)	19.93 (22.02)	62.73 (65.22)	92.67 (93.53)	99.50 (99.58)	99.99 (99.99)	100.0 (100.0)	100.0 (100.0)	100.0 (100.0)
	400	**5.05 **(**5.09**)	36.11 (40.14)	90.44 (92.05)	99.86 (99.92)	100.0 (100.0)	100.0 (100.0)	100.0 (100.0)	100.0 (100.0)	100.0 (100.0)
										
0.4	50	-	-	-	-	-	-	-	-	-
	100	**5.87 **(**5.17**)	7.91 (9.77)	21.92 (25.30)	45.15 (48.55)	70.62 (72.95)	93.44 (88.64)	98.29 (96.46)	99.63 (99.13)	99.96 (99.82)
	200	**5.59 **(**5.13**)	11.58 (16.32)	40.43 (47.45)	76.71 (81.23)	95.15 (96.48)	99.88 (99.58)	100.0 (99.99)	100.0 (100.0)	100.0 (100.0)
	400	**5.84 **(**4.76**)	19.00 (28.10)	68.88 (77.88)	98.88 (98.36)	99.93 (99.98)	100.0 (100.0)	100.0 (100.0)	100.0 (100.0)	100.0 (100.0)
										
0.6	50	-	-	-	-	-	-	-	-	-
	100	-	-	-	-	-	-	-	-	-
	200	**6.79 **(**4.71**)	6.75 (10.73)	20.87 (28.78)	48.56 (57.07)	77.02 (81.95)	98.53 (93.71)	99.81 (98.52)	99.99 (99.76)	100.0 (99.99)
	400	**8.42 **(**4.48**)	8.88 (18.17)	38.42 (54.88)	78.94 (88.15)	97.28 (98.78)	99.99 (99.91)	100.0 (100.0)	100.0 (100.0)	100.0 (100.0)
										
0.8	50	-	-	-	-	-	-	-	-	-
	100	-	-	-	-	-	-	-	-	-
	200	-	-	-	-	-	-	-	-	-
	400	**10.95 **(**4.40**)	5.22 (10.97)	17.99 (33.05)	48.43 (63.97)	79.65 (87.84)	99.88 (96.46)	99.99 (99.35)	100.0 (99.95)	100.0 (100.0)

Percentage of the number of associations (rejected hypothesis) obtained using standard OLS and corrected OLS (between brackets) in 10,000 simulations. The first line contains the strength of association between predictors and response variables as described in simulation I. The rate of false-positives was controlled in 5%. In bold, are the rate of false-positives, i.e., the number of false-positives divided by the number of simulations. "-" means that it was not possible to calculate due to high measurement error when compared to sample's size. EM: Standard deviation of the Error of Measure. n: Number of samples. Notice that, in fact, the corrected OLS controls the rate of false positives in 5% while the usual OLS does not (values in bold).

**Figure 1:**
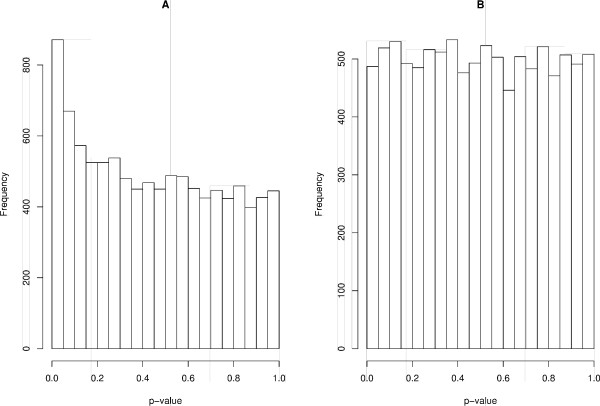
**P-value distribution under the null hypothesis (*β*_1 _= 0) in independent data with standard deviation of the measurement error equal to 0.6 and sample size equal to 400 (model described in Simulations section, simulation I)**. This simulation was performed 10,000 times. (A) Standard Ordinary Least Squares (non uniform distribution); (B) Corrected Ordinary Least Squares (uniform distribution).

Analyzing Figure 1A, we conclude that, the standard OLS is not controlling the rate of false positives in the presence of measurement error for any significance level (p-value threshold). On the other hand, Figure 1B describes the consistency of the test performed by the corrected OLS, i.e., the uniform distribution of p-values illustrates that the rate of false positives is actually controlled under any considered threshold, since the uniform distribution emerges for p-values when the distribution of the statistic is correctly specified (otherwise, the p-value distribution may not be uniform).

**Figure 2:**
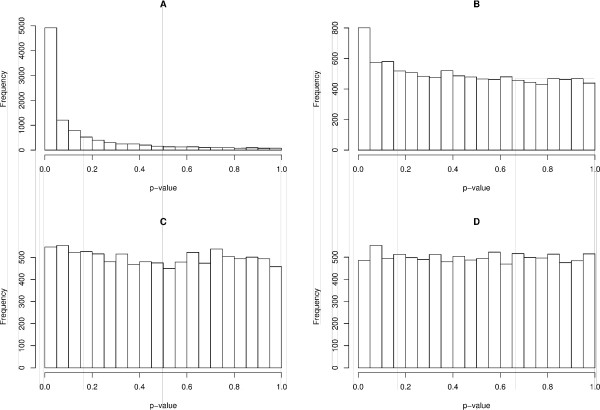
**P-value distribution under the null hypothesis in time series data with standard deviation of the measurement error equal to 0.6 and time series length equal to 400 (model described in Simulations section, simulation II)**. This simulation was performed 10,000 times. (A) Standard VAR p-value distribution of autoregressive coefficient *β*_0 _= 0 (non uniform distribution); (B) Standard VAR p-value distribution of cross-autoregressive coefficient *β*_1 _= 0 (non uniform distribution); (C) Corrected VAR p-value distribution of autoregressive coefficient *β*_0 _= 0 (uniform distribution); (D) Corrected VAR p-value distribution of cross-autoregressive coefficient *β*_1 _= 0 (uniform distribution).

**Figure 3:**
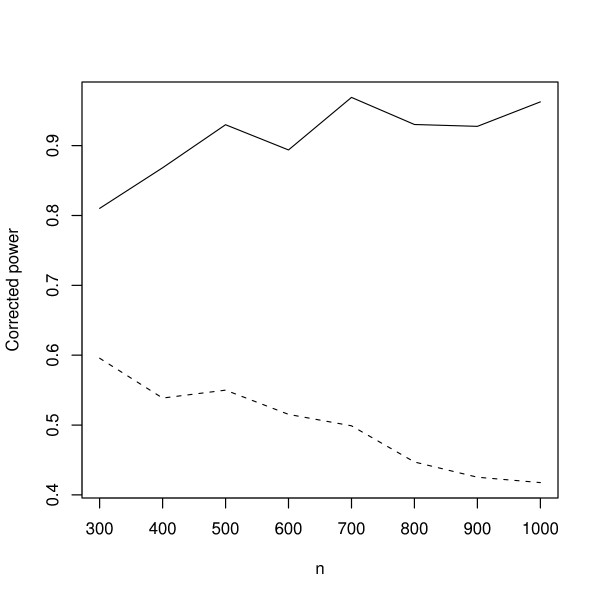
**Corrected power curve**. The full line represents the proposed OLS and the dashed line represents the standard OLS. It was performed 15,000 Monte Carlo simulations (model described in simulation I) for each *n *where *n *varied from 300 to 1,000 in steps of 100. *n*: sample size. P-value and standard deviation of measurement error were set to 0.05 and 0.5, respectively.

**Figure 4:**
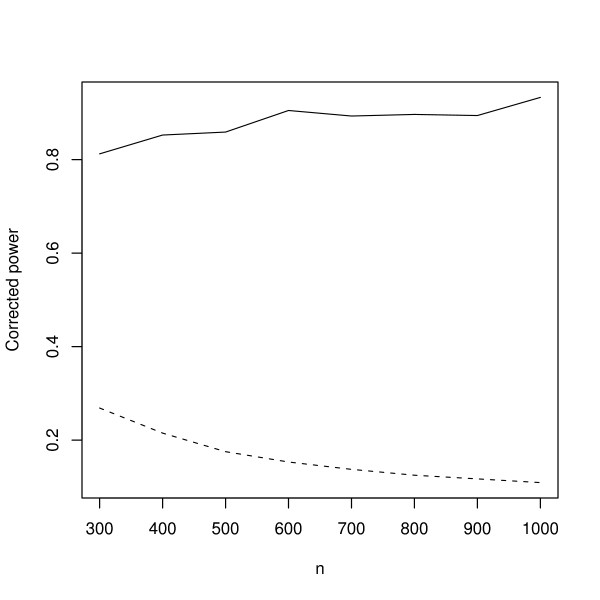
**Corrected power curve**. The full line represents the proposed VAR and the dashed line represents the standard VAR. It was performed 15,000 Monte Carlo simulations (model described in simulation II) for each *n *where *n *varied from 300 to 1,000 in steps of 100. *n*: sample size. P-value and standard deviation of measurement error were set to 0.05 and 0.5, respectively.

In the time series case, similar results were observed. The standard VAR estimates produce biased coefficients in the presence of measurement error (Table 3). Moreover, there is no control of the type I error in both, autoregressive and cross-autoregressive coefficients (in all the text, in order to simplify the notation, autoregressive coefficient will denote the auto-loop, i.e., the coefficient related to *z*_*i*, *t*-*r *_→ *z*_*i*, *t *_and cross-autoregressive coefficient will represent the coefficient for *z*_*j*, *t*-*r *_→ *z*_*i*, *t*_, where *i *≠ *j *and *r *<*t*) (Table 4). Analyzing the results produced by the proposed VAR model (Table 3 - values between brackets), it is possible to observe that the estimated coefficients converge to the true value as time series length increases. Notice that, the results produced by the standard VAR model indicate that, increasing the sample size does not imply in convergence of the estimates to the true values (Table 3). By observing Table 4, we see that the corrected VAR approach is actually controlling the rate of false positives in the set significance level (p < 0.05). Figure 2 emphasizes this result. Figure 2A and 2B describe the p-value distributions of autoregressive and cross-autoregressive coefficients of standard VAR under the null hypothesis (*β*_0 _= 0 (autoregressive) and *β*_1 _= 0 (cross-autoregressive)). Notice that when *β*_0 _= *β*_1 _= 0, the p-value distributions should be uniform in the interval [0,1]. However, there is a high concentration around zero, demonstrating that the rates of false positives are inflated (and consequently not controlled) in both autoregressive or cross-autoregressive cases. In Figures 2C and 2D, the p-value distributions are uniform, i.e., the test under the null hypothesis (*β*_0 _= 0 and *β*_1 _= 0) using the corrected VAR model is actually controlling the type I error in autoregressive and cross-autoregressive coefficients (uniform distribution). Figures 3 and 4 illustrates the corrected power curves for both, OLS and VAR. The corrected power curve *P*^*c*^(*α*) can be defined as(1)

**Table 3:** Vector autoregressive model.

EM	n	***β***_**0**_	***β***_**1**_	***β***_**2**_	***β***_**3**_	***β***_**4**_	***β***_**5**_	***β***_**6**_	***β***_**7**_	***β***_**8**_	***β***_**9**_
		0	0	-0.1	-0.2	-0.3	-0.4	0.5	0.6	0.7	0.8
											
0	50	-0.04	0.00	-0.10	-0.20	-0.30	-0.41	0.51	0.61	0.71	0.81
	100	-0.02	0.00	-0.10	-0.20	-0.30	-0.40	0.50	0.61	0.71	0.80
	200	-0.01	0.00	-0.10	-0.20	-0.30	-0.40	0.50	0.60	0.70	0.80
	400	0.00	0.00	-0.10	-0.20	-0.30	-0.40	0.50	0.60	0.70	0.80
											
0.2	50	-0.03 (-0.04)	0.01 (0.00)	-0.09 (-0.10)	-0.19 (-0.21)	-0.28 (-0.31)	-0.38 (-0.42)	0.49 (0.51)	0.58 (0.61)	0.69 (0.72)	0.78 (0.82)
	100	-0.01 (-0.02)	0.00 (0.00)	-0.09 (-0.10)	-0.19 (-0.20)	-0.28 (-0.31)	-0.38 (-0.41)	0.48 (0.50)	0.58 (0.61)	0.68 (0.71)	0.78 (0.81)
	200	0.00 (-0.01)	0.00 (0.00)	-0.09 (-0.10)	-0.19 (-0.20)	-0.28 (-0.30)	-0.38 (-0.40)	0.49 (0.50)	0.58 (0.60)	0.68 (0.71)	0.77 (0.81)
	400	0.01 (0.00)	0.00 (0.00)	-0.09 (-0.10)	-0.19 (-0.20)	-0.28 (-0.30)	-0.38 (-0.40)	0.48 (0.50)	0.58 (0.60)	0.68 (0.70)	0.77 (0.80)
											
0.4	50	- (-)	- (-)	- (-)	- (-)	- (-)	- (-)	- (-)	- (-)	- (-)	- (-)
	100	0.02 (-0.03)	0.02 (0.00)	-0.07 (-0.11)	-0.16 (-0.21)	-0.24 (-0.32)	-0.32 (-0.42)	0.44 (0.52)	0.53 (0.62)	0.61 (0.72)	0.70 (0.83)
	200	0.03 (-0.01)	0.02 (0.00)	-0.07 (-0.10)	-0.16 (-0.21)	-0.24 (-0.31)	-0.32 (-0.41)	0.44 (0.51)	0.53 (0.61)	0.61 (0.71)	0.70 (0.81)
	400	0.04 (-0.01)	0.02 (0.00)	-0.07 (-0.10)	-0.15 (-0.20)	-0.24 (-0.30)	-0.33 (-0.41)	0.44 (0.50)	0.53 (0.61)	0.61 (0.71)	0.70 (0.81)
											
0.6	50	-	-	-	-	-	-	-	-	-	-
	100	-	-	-	-	-	-	-	-	-	-
	200	0.06 (-0.02)	0.03 (-0.01)	-0.04 (-0.11)	-0.12 (-0.21)	-0.19 (-0.32)	-0.26 (-0.42)	0.39 (0.52)	0.46 (0.62)	0.53 (0.73)	0.60 (0.83)
	400	0.07 (-0.01)	0.03 (0.00)	-0.04 (-0.10)	-0.12 (-0.20)	-0.19 (-0.31)	-0.26 (-0.41)	0.39 (0.51)	0.46 (0.61)	0.53 (0.71)	0.60 (0.81)
											
0.8	50	-	-	-	-	-	-	-	-	-	-
	100	-	-	-	-	-	-	-	-	-	-
	200	-	-	-	-	-	-	-	-	-	-
	400	0.10 (-0.02)	0.04 (0.00)	-0.02 (-0.11)	-0.08 (-0.21)	-0.14 (-0.32)	-0.20 (-0.42)	0.33 (0.52)	0.39 (0.62)	0.45 (0.72)	0.51 (0.83)

Standard VAR average estimated coefficients and corrected VAR (between brackets) in 10,000 simulations. The first line contains the strength of association between predictors and response variables as described in simulation II. "-" means that it was not possible to calculate due to high measurement error when compared to sample's size. EM: Standard deviation of the Error of Measure. n: Number of samples. Notice that, in the presence of measurement error, the coefficients (*β*) estimated by the corrected VAR (between brackets) converge to the "true" values, while the estimated by standard VAR do not.

**Table 4:** Vector autoregressive model.

EM	n	***β***_**0**_	***β***_**1**_	***β***_**2**_	***β***_**3**_	***β ***_**4**_	***β***_**5**_	***β***_**6**_	***β***_**7**_
		0	0	-0.1	-0.2	-0.3	-0.4	0.5	0.6
									
0	50	**6.48**	**5.66**	10.47	23.78	44.69	66.62	83.62	93.13
	100	**5.86**	**5.44**	16.09	49.39	81.50	96.45	99.37	99.97
	200	**5.72**	**5.07**	30.90	81.55	98.90	100.00	100.00	100.00
	400	**5.19**	**5.21**	54.80	98.49	100.00	100.00	100.00	100.00
									
0.2	50	**5.39 **(**6.64**)	**5.08 **(**5.40**)	8.13 (8.80)	20.17 (20.78)	37.99 (38.41)	59.73 (59.74)	78.68 (75.96)	89.67 (87.72)
	100	**5.26 **(**6.17**)	**5.20 **(**5.20**)	13.41 (14.65)	41.15 (42.79)	74.12 (75.29)	93.39 (93.78)	98.84 (98.60)	99.91 (99.91)
	200	**4.84 **(**5.50**)	**5.22 **(**5.46**)	24.19 (26.85)	73.89 (76.10)	97.15 (97.57)	99.91 (99.92)	100.0 (100.0)	100.0 (100.0)
	400	**5.72 **(**5.09**)	**5.53 **(**5.44**)	44.73 (48.95)	96.59 (97.29)	99.97 (99.98)	100.0 (100.0)	100.0 (100.0)	100.0 (100.0)
									
0.4	50	-	-	-	-	-	-	-	-
	100	**6.02 **(**6.48**)	**5.03 **(**5.15**)	9.59 (11.39)	27.88 (31.93)	54.11 (57.93)	79.25 (81.32)	95.92 (93.17)	99.12 (98.03)
	200	**10.20 **(**5.79**)	**5.45 **(**4.97**)	14.37 (19.86)	51.19 (58.67)	86.74 (90.40)	98.45 (98.90)	99.96 (99.88)	100.0 (100.0)
	400	**20.49 **(**5.52**)	**5.64 **(**5.06**)	25.14 (36.21)	82.24 (88.42)	99.29 (99.66)	99.98 (100.0)	100.0 (100.0)	100.0 (100.0)
									
0.6	50	-	-	-	-	-	-	-	-
	100	-	-	-	-	-	-	-	-
	200	**22.79 **(**5.39**)	**5.98 **(**5.14**)	8.13 (13.65)	29.25 (39.74)	61.55 (70.80)	87.76 (91.46)	99.65 (98.34)	99.93 (99.68)
	400	**49.01 **(**5.36**)	**7.56 **(**4.97**)	12.29 (24.33)	52.77 (69.83)	90.96 (95.68)	99.52 (99.83)	100.0 (100.0)	100.0 (100.0)
									
0.8	50	-	-	-	-	-	-	-	-
	100	-	-	-	-	-	-	-	-
	200	-	-	-	-	-	-	-	-
	400	**70.58 **(**5.49**)	**9.73 **(**5.29**)	6.26 (15.25)	27.24 (46.89)	65.41 (81.53)	91.82 (96.48)	99.98 (99.45)	100.0 (99.45)

									

**EM**	**n**	***β***_**8**_	***β***_**9**_						

		0.7	0.8						
									
0	50	97.52	99.20						
	100	100.00	100.00						
	200	100.00	100.00						
	400	100.00	100.00						
									
0.2	50	96.11 (94.87)	98.44 (98.89)						
	100	100.0 (99.99)	100.0 (100.0)						
	200	100.0 (100.0)	100.0 (100.0)						
	400	100.0 (100.0)	100.0 (100.0)						
									
0.4	50	-	-						
	100	99.89 (99.65)	99.99 (99.97)						
	200	100.0 (100.0)	100.0 (100.0)						
	400	100.0 (100.0)	100.0 (100.0)						
									
0.6	50	-	-						
	100	-	-						
	200	100.0 (100.0)	100.0 (100.0)						
	400	100.0 (100.0)	100.0 (100.0)						
									
0.8	50	-	-						
	100	-	-						
	200	-	-						
	400	100.0 (99.99)	100.0 (100.0)						

Percentage of the number of associations (rejected hypothesis) obtained using standard VAR and corrected VAR (between brackets) in 10,000 simulations. The first line contains the strength of association between predictors and response variables as described in simulation II. The rate of false-positives was controlled in 5%. In bold, are the rate of false-positives, i.e., the number of false-positives divided by the number of simulations. "-" means that it was not possible to calculate due to high measurement error when compared to sample's size. EM: Standard deviation of the Error of Measure. n: Number of samples. Notice that, in fact, the corrected VAR controls the rate of false positives in 5% in both cases, autoregressive (*β*_0_) and cross-autoregressive (**β**_1_) coefficients, while the usual VAR does not (values in bold).

where *α *is the adopted type I error nominal level, *P*(*a*(*α*)) is the power using the true probability of the type I error, namely *a*(*α*). Notice that the corrected power is just the power penalized by the distance between *a*(*α*) and *α*. This correction in the power is necessary because under the null hypothesis, the power has to be the nominal level, and for comparing powers from different statistics it must be done using the same nominal level.

For a good statistic, notice that under an alternative hypothesis and when *n *→ ∞, the corrected power *P*^*c*^(*α*) converges to one because  and . On the one hand, for a statistic that does not control the rate of false positives, for example, when *α *is set to 5% and the true probability of the type I error is *a*(*α*) = 0.08, since *a*(*α*)/*α *is greater than one, *P*^*c*^(*α*) will not converge to one. On the other hand, for a good statistic, the rate *a*(*α*)/*α *converges to one when *n *→ ∞, then *P*^*c*^(*α*) will converge to one. Analyzing Figures 3 and 4, it is possible to verify that, for standard OLS and VAR approaches (dashed lines), the ratio *a*(*α*)/*α *increases faster than the corresponding powers *P*(*a*(*α*)), i.e., the dashed lines is decreasing as *n *increases. Notice on Tables 2 and 4 that the rates of false positives (*a*(*α*)) increase as *n *increases, and consequently, in our specific case, the ratio *a*(*α*)/*α *increases and the corrected power *P*^*c*^(*a*(*α*)) converges to zero. On the other hand, the proposed methods (full lines) keep the false positive rates controlled while the corrected power increases as *n *increases. It can be observed by the full lines converging to one (Figures 2 and 4) and also on Tables 2 and 4. The variations present in the curves are probably due to variations in Monte Carlo simulations, since these variations decreased (become smoother) when the number of simulations was increased from 5,000 to 10,000 and from 10,000 to 15,000. In order to illustrate the performance of standard and corrected OLS and VAR approaches in actual biological data, firstly, the measurement error was estimated using the method described in the *Measurement error estimation *section (*No technical replicates *subsection). Then, the *TP53 *network was constructed using a dataset composed of 400 microarrays.

Table 5 illustrates the results of a multivariate regression using OLS. Four genes known to be direct targets of *TP53 *were selected, namely, *MDM2*, *FAS*, *BAX *and *MAP4*, and a multivariate network was constructed using OLS. In fact, these four genes were actually identified as targets of *TP53 *(high t-statistics). Notice that comparing the standard and corrected OLS estimators, it is possible to conclude that the t-statistics are different probably due to the biased standard OLS estimator in the presence of measurement error.

**Table 5:** Gene TP53 (lung cancer data).

Association	*t*(*β*_***standard***_)	*t*(*β*_***corrected***_)	*t*(*β*_***standard***_) -*t*(*β*_***corrected***_)
p53 → mdm2	-2.2550	-2.1250	-0.1299
p53 → fas	-3.3547	-3.0059	-0.3487
p53 → bax	5.2148	4.5290	0.6859
p53 → map4	2.8486	3.0243	-0.1757
			
mdm2 → fas	-1.5495	-1.5002	-0.0493
mdm2 → bax	0.1880	0.4716	-0.2836
mdm2 → map4	-0.8153	-0.2766	-0.5387
			
fas → bax	0.0987	0.5746	-0.4759
fas → map4	2.5776	2.6374	-0.0598
			
bax → map4	-0.3538	-0.7187	0.3650

; . The direction of the arrows means the direction of regression, i.e., in the head of the arrow is the predictor and in the tail of the arrow is the response variable. Since it is not a time series data, the *t *statistics are equal independent of the direction of regression, in other words, the *t *statistics of *x *→ *y *or *y *→ *x *are equal.

Table 6 shows the application of both, standard and proposed VAR models in a set of well known five genes related to circadian rhythm, namely, *CLOCK*, *CRY2*, *PER2*, *PER3 *and *DBP*. The genes *CRY2*, *PER2*, *PER3 *and *DBP *are known to be regulated by the complex BMAL1-CLOCK in mammals [24]. A VAR process of order one was adjusted and applied in a multivariate manner. Notice that also in the time series data, the estimators presented different results due to measurement error.

**Table 6:** Gene CLOCK (actual data).

Association	*t*(*β*_***standard***_)	*t*(*β*_***corrected***_)	*t*(*β*_***standard***_) -*t*(*β*_***corrected***_)
clock → clock	-2.5462	-2.3086	-0.2376
clock → cry2	1.4255	1.4165	0.0090
clock → per2	-0.1459	0.2372	-0.3830
clock → per3	0.5827	0.5320	0.0507
clock → dbp	-1.6838	-1.6204	-0.0634
			
cry2 → clock	-0.8201	-0.9070	0.0869
cry2 → cry2	-3.0326	-2.9813	-0.0513
cry2 → per2	0.7007	-0.0915	0.7921
cry2 → per3	0.8740	0.5134	0.3606
cry2 → dbp	0.5087	0.7566	-0.2479
			
per2 → clock	2.3427	2.3032	0.0394
per2 → cry2	0.8596	0.9123	-0.0527
per2 → per2	-1.7977	-1.6259	-0.1718
per2 → per3	-0.4415	-0.5319	0.0904
per2 → dbp	-0.7264	-0.7320	0.0056
			
per3 → clock	-1.3426	-1.3651	0.0225
per3 → cry2	-0.0824	-0.0569	-0.0255
per3 → per2	0.0492	-0.0515	0.1007
per3 → per3	-1.9925	1.6944	-3.6869
per3 → dbp	0.4787	0.4176	0.0611
dbp → clock	0.1788	0.1420	0.0368
			
dbp → cry2	0.4228	0.3759	0.0469
dbp → per2	1.0039	0.8547	0.1492
dbp → per3	-0.6207	-0.2896	-0.3311
dbp → dbp	-1.0694	-1.1063	0.0369

;

Comparison of the usual and proposed methods in actual biological data is a difficult task since no one knows the "true" values. However, as observed in the simulation results, it is possible to conclude that the corrected approaches provide more reasonable results than biased standard methods.

In order to uncover more details about the performance of both, OLS and VAR, other experiments were conducted. These experiments consist in adding correlation in the residues and testing other null hypothesis (data not shown). The results obtained ignoring the errors by these methods can be compiled as:

1. in both, independent and time series data, standard OLS does not work correctly in the presence of measurement error and correlated residues;

2. in the presence of measurement error and no correlation among all predictors of independent data, the t-test built, under the standard OLS approach, to test *H*_0 _: *β*_*j *_= *m *for *j *= 1,..., *p *works perfectly only if *m *= 0 (for other null hypothesis this t-test does not work correctly). This happens because, under this hypothesis, there is no covariate effect and, consequently, there is no measurement error effect associated with the covariate. The same behavior can be seen in Patriota *et al*. (2009) [25];

3. in the time series case, the t-test (or Wald's test) does not control the type I error rate in the presence of measurement error, independent whether there is or not correlation between time series;

4. in the presence of measurement error, the estimates obtained by standard OLS are always attenuated.

Therefore, these results demonstrate that improved methods to construct regulatory networks become necessary, since it is known that genes belong to an intrincate network, i.e., the covariates may be correlated and, moreover, gene expression quantification processes such as microarray technology measure with considerable error. If these conditions are ignored, one may obtain distort results and, consequently, conclude that there is a relationship between gene expressions where there is no association.

Construction of large networks is a challenge in bioinformatics. The methods proposed here do not allow the identification of networks when the number of variables is larger than the number of observations. Increasing the number of variables, the estimates become imprecise and the chances of obtaining multicollinearity problems also increases. In the presence of multicollinearity, one may use a feature selection procedure such as a stepwise (forward or backward, for example) in order to choose the optimum set of predictors.

Analyzing Pearson correlation coefficient, one can observe that it is simply a normalized linear regression coefficient (OLS) between -1 and 1. Therefore, Pearson correlation-based methods such as Relevance networks [14] or Graphical Gaussian models [26] need further studies in order to evaluate if they are also super-estimating the rate of false positives and attenuating the coefficients like OLS. Moreover, Pearson correlation is widely used in order to test linear correlation between a certain gene expression signal and another characteristic such as prognostic, phenotype, tumor grade etc. Since these covariates may be measured with error, it is also crucial to develop a corrected Pearson correlation.

In order to develop a corrected Pearson correlation for measurement error, verify that it is possible to use the improved OLS presented in this report. The corrected Pearson correlation (*ρ*) between two random variables *X *and *Y*, both measured with error is given by(2)

where *β *should be estimated by using the corrected OLS (i.e., by simultaneously considering the three equations: *y *= *α *+ *βx *+ *ε*, *X *= *x *+ ϵ_1 _and *Y *= *y *+ ϵ_2_), *σ*_*X *_and *σ*_*Y *_are the standard deviations of the observed variables *X *and *Y*, respectively, and  and  are the standard deviations of the error of measure ϵ_1 _and ϵ_2_, respectively. In this way, the estimate of the corrected version of the Pearson correlation is consistent (the larger the sample size, the smaller estimation error tends to be). Notice that, the difference between the corrected and uncorrected version of the Pearson correlation is that we are removing the excess of variability from the estimated variances of the latent *x *and *y*, since the sample variances of *X *and *Y *always over-estimate them due to the measurement errors ϵ_1 _and ϵ_2 _(note that,  and ), where  and  are the variances of *x *and *y*, respectively.

Although the examples provided here only treat regulatory network models, the proposed approaches can be applied in a straightforward manner also to estimate linear relationships between random variables measured with error.

## Conclusions

Unfortunately, avoiding measurement error in a complete manner is a very difficult task, however, it can be minimized in the measuring (experimental) process and treated during the data analysis step. Here, we have shown evidence that presence of the measurement errors has a high impact in regulatory network models. In order to overcome this problem, approaches in both major data conditions, independent and time series data were proposed in addition to measurement error estimation procedures. Further studies are necessary in order to verify how is the performance of other regulatory networks (Bayesian networks, Structural Equation models, Graphical Gaussian models, Relevance networks, etc) in the presence of measurement error.

## Methods

In this section, standard Ordinary Least Squares and Vector Autoregressive models will be described. Furthermore, corrected methods for measurement error will also be presented. Finally, the model used in the simulations will be detailed.

### Ordinary least squares

In a multivariate regression model, let *x*_1_, *x*_2_,..., *x*_*p *_be *p *predictor variables (genes) possibly being related to a response variable *y *(gene). The conventional linear regression model states that gene *y *is composed of an intercept or constant *a *which is the basal expression level of *y*, the predictors or gene expressions *x*_*j *_'s (*j *= 1,..., *p*) which relationship with *y *is represented by *β *= (*β*_1_,..., *β*_*p*_)^⊤ ^(the sign of *β*_*j *_represents the relationship between *y *and *x*_*j*_, i.e., positive or negative association), and a random error *ε*, which accounts for an intrinsic biological variation (this is not the measurement error).

With *n *independent observations (microarrays) *y *and the associated gene expression values of *x*_*j*_, the complete model becomes

for *i *= 1,..., *n*. In matrix notation, it is described as(3)

where(4)

The entire vector of error terms, *ε*_*i *_= (*ε*_*i*1_,..., *ε*_*iq*_)^⊤^, are assumed to be independent and identically distributed as a *q*-variate normal distribution with zero vector mean and positive definite covariance matrix **Σ**_*ε *_for all *i *= 1,..., *n*, where *q *is the number of response variables. Notice that the proposed method is considering the homoscedastic case, i.e., the covariance matrix **Σ**_*ε *_does not change with *i*. Let **Σ**_*yx*_, **Σ**_*xx *_and **Σ**_*yy *_be the covariances of (*y, x*), (*x, x*) and (*y, y*), respectively. These covariance matrices could be estimated by:(9)

and(11)

where(12)

and(13)

Then, the intercept *α *is estimated as(14)

and the estimator for the model's coefficient is given by(15)

The asymptotic variance-covariance matrix of vec() and its estimate are given, respectively, by(16)

and(17)

where ⊗ is the Kronecker product and  (non biased estimator). Notice that, the diagonal elements of  are the variances of the elements of , say  for *j *= 1,..., *p*.

Let  denote the fitted values of *y*, then, the residuals are(18)

#### Hypothesis testing

The main interest in a simple regression model (**y**_*i *_= *α *+ *β***x**_*i *_+ *ε*_*i*_) lies in testing the strength of the relationship between the predictor variable (gene) *x *and the response variable (gene) *y*, in other words, if *β *is equal to a certain value *m *(in general, *m *= 0, i.e., there is or not linear relationship between genes *x *and *y*).

The asymptotic distribution of vec() is given by(19)

and the test is described by:

This test may be performed using the Wald-type statistic expressed as(20)

where **C **is a matrix of contrasts (usually, **C **= **I**). For more details about the matrix of contrasts, see [27]. Under the null hypothesis, (20) has a limit *χ*^2^(*d*) distribution, where *d *= *rank*(**C**) gives the number of linear restrictions.

### Ordinary least squares with measurement error

Now, we shall study models of the regression type where one is unable to observe expression values of genes *x *and *y *(as described before) directly. Instead of observing *x *and *y*, one observes the sum(21)

and(22)

with(23)

where ϵ_1 _~ *N*(**0**, ) independent of ϵ_2 _~ *N*(**0**, ) with  and  known are called as measurement errors, i.e., the variation derived by the measurement process (for example, the measurement error introduced when analyzing microarrays), *ε *~ *N*(**0**, **Σ**_*ε*_) is the random error (intrinsic biological variation) and **x **~ *N *(*μ*_*x*_, **Σ**_*xx*_), **y **~ *N*(*μ*_*y*_, **Σ**_*yy*_) with *μ*_*y *_= *α *+ *βμ*_*x *_and **Σ**_*yy *_= *β***Σ**_*xx*_*β*^⊤ ^+ **Σ**_ε_.

The matrices  and  are given by(24)

and(25)

i.e., the measurement errors may be different for each variable. Notice that the components of the measurement error's vector may be correlated but the entire vectors are independent.

Let **Σ**_*YX*_, **Σ**_*XX *_and **Σ**_*YY *_be the sample covariances of (*Y, X*), (*X, X*) and (*Y, Y*), respectively. These covariance matrices could be estimated by substituting *x *and *y *by *X *and *Y *in equations (8-12). Then, the intercept *a *is estimated as(26)

and the estimator for the model's coefficient is given by(27)

where(28)

Notice that  is estimated using equation (10) and  must be known *a priori *(it can be estimated using the procedures described in the section "Measurement errors estimation").

The asymptotic variance-covariance matrix of vec() and its estimate are given, respectively, by (the proof is in the Appendix)(29)

and(30)

where **I**_*q *_denotes the *q *× *q *identity matrix and(31)

Notice that, in the absence of measurement error, i.e.,  the corrected OLS is exactly equal to standard OLS. Furthermore, it is noteworthy that this asymptotic variance is similar to the one presented by [23] but in a multivariate manner with no correlation in the errors.

#### Hypothesis testing

Similar to the OLS with no measurement error, the interest in a simple regression model (**y**_*i *_= *α *+ *β***x**_*i *_+ *ε*_*i*_) lies in testing the strength of the relationship between the predictor gene *x *and the response gene *y*. The asymptotic distribution of vec() is given by(32)

and the test is similar to the previous case (standard OLS) described by:

This test may be performed using the Wald-type statistic expressed as(33)

where **C **is a matrix of contrasts. Under the null hypothesis, (33) follows a *χ*^2 ^distribution with *rank*(**C**) degrees of freedom.

### Vector autoregressive model

Here we define the usual VAR model as defined in Lütkepohl (2006) [28].

Let **z**_*t *_= (*z*_1*t*_,..., *z*_*pt*_)^⊤ ^be a (*p *× 1) vector of time series variables. The usual VAR(*r*) model (of order *r*) has the form(34)

where *n *is the time series length, *β*_*j *_for *j *= 1,..., *p *are (*p *× *p*) coefficient matrices and *ε*_*t *_is an (*p *× 1) unobservable zero mean white noise vector process with covariance matrix **Σ**_*ε*_. Under stationarity conditions, the mean and autocovariance function are given, respectively, by(35)

and(37)

where **I**_*p *_denotes the *p *× *p *identity matrix.

The model (34) can be re-written as(38)

where *β *= (*β*_1_*β*_2_... *β*_*r*_) is a *p *× *pr *matrix and .

Therefore, if the white noise (*ε*) has normal distribution, the conditional Maximum Likelihood (ML) estimators of *α*, *β *and **Σ**_*ε *_are equal to the OLS estimators. They are given, respectively by(39)

and(41)

where(42)

and(46)

The consistence of those conditional ML estimators is assured under the stationary conditions [28]. The covariance function of  is given by(47)

### Vector autoregressive model with measurement error

Now, the VAR model with measurement error will be presented.

Let **z**_*t *_be the "true" variables that are not directly observed. Let **Z**_*t *_be the observed surrogate variables which have an additive structure given by(48)

where **Z**_*t *_= (*Z*_1*t*_, *Z*_2*t*_,..., *Z*_*pt*_)^⊤ ^is the surrogate vector and ϵ_*t *_= (ϵ_1*t*_, ϵ_2*t*_,..., ϵ_*pt*_)^⊤ ^is the measurement error vector. In most cases, if the usual conditional ML estimator is adopted for the observations subject to errors, i.e., replacing **z**_*t *_with **Z**_*t *_in the equation (34), the estimator of *β *will be biased as well as its asymptotic variance. Therefore, in order to overcome this limitation the measurement errors should be included in the estimation procedure. Nevertheless, the model (34) plus the equation (48) is not identifiable, since the covariance matrices of *ε*_*t *_and ϵ_*t *_are confounded. This problem can be avoided considering known the variance of ϵ_*t*_.

Let ϵ_*t *_~ *N *(0, **Σ**_ϵ_) be the measurement error with **Σ**_ϵ _known (refer to section *Measurement error estimation *for details about how to estimate **Σ**_ϵ_). Then, the parameters of the model (34) under measurement errors as in (48) have consistent estimators (Patriota *et al*.: Vector autoregressive models with measurement errors for testing Granger causality, submitted) given by(49)

and(51)

where(52)

Then, the asymptotic distribution of vec() is given by [29](55)

where the matrix  is given by(56)

where

where **Σ**_*v *_= **Σ**_*ε *_+ **Σ**_ϵ _+ *β*(**I**_*r *_⊗ **Σ**_ϵ_)*β*^⊤ ^and **J**_*l *_is a (*r *× *r*) matrix of zeros with one's in the |*l*|^*th *^diagonal above (below) the main diagonal if *l *> 0 (*l *< 0) and **J**_0 _is a (*r *× *r*) matrix of zeros.

Notice that, if *r *= 1 we have the VAR(1) model and the asymptotic covariance simplifies to(57)

where(58)

The *i*^*th *^element of vec(), is asymptotically normally distributed with standard error given by the square root of *i*^*th *^diagonal element of . Thus, we can construct hypotheses testing on the individual coefficients, or in more general form of contrasts

involving coefficients across different equations of the VAR model. It may be tested using the Wald statistic conveniently expressed as(59)

where **C **is a matrix of contrasts (**C **= **I**, for instance) and **m **is usually a (*p *× 1) vector or zeros.

Under the null hypothesis, (59) has a limiting *χ*^2^(*d*) distribution where *d *= rank(**C**) gives the number of linear restrictions. This test is useful to identify, in a statistical sense (controlling the rate of false positives), which gene (predictor variable) is Granger causing another gene (response variable).

### Measurement error estimation

Here, two methods to estimate measurement error are proposed. One when technical replicates are available and another one in the case when they are not available.

#### Technical replicates

When technical replicates are available, measurement error estimation may be performed by applying a strategy extending the methods described by Dahlberg (1940) [30] (more details about Dahlberg's method in the Appendix). For microarray data, it is known that the variance varies along the spots (heteroscedasticity) due to variations in experimental conditions (efficiency of dye incorporation, washing process, etc) [31]. Moreover, it is known that Dahlberg's approach is not suitable in the presence of systematic errors. Therefore, the application of the Dahlberg's formula is not straightforward. In order to overcome this problem, we suggest the following algorithm [32].

Let *W *and *W' *be two microarrays, where *W' *is the technical replicate of *W*.

1. Perform a non-linear regression such as splines smoothing between *log*(*W*) and *log*(*W'*), i.e., *log*(*W'*) = *f*(*log*(*W*)) + *ε*_1_. Notice that the logarithm was calculated as a variance stabilizer (due to the high variance observed in microarray data). This is a common practice in microarray analysis;

2. Apply again the splines smoothing between  and *log*(*W*), i.e.,  = *g*(*log*(*W*)) + *ε*_2_;

3. Calculate . This is a possible estimate for the standard deviation of the measurement error. Notice that with this process, we obtain one  for each spot *i *= 1,..., *m*, where *m *is the number of spots in the microarray, also in the presence of heteroscedasticity.

#### No technical replicates

However, unfortunately, technical replicates is not always available. To this case, we have developed a strategy based on negative control probes and housekeeping genes frequently provided in commercial microarrays. Technically, housekeeping genes and negative controls should not change their expression levels [33]. Therefore, any variation measured by them can be understood as measurement error. In order to overcome the problem of heteroscedasticity in microarrays, we present a method based on splines smoothing. The main idea of this method consists in estimating how much of the total variance (intrinsic biological variation + measurement error) is due to measurement error. The method is as follows:

1. Let *S *be the set of all probes in the microarray and *H *be the set of housekeeping genes and negative controls. Calculate the mean and variance for each probe of *S *and *H*;

2. Perform a splines smoothing in both sets of probes separately, i.e., a splines smoothing *var*(*H*) = *f*(*mean*(*H*)) + *ε*_1 _and *var*(*S*\{*H*}) = *g*(*mean*(*S*\{*H*})) + *ε*_2_, where *H *is a matrix containing the expression values of each housekeeping gene and negative controls in each row and *S*\{*H*} is a matrix containing the expression values of the remaining set of probes in each row. The functions *f *and *g *may be represented by a linear combination of spline functions *ϕ*_*j*_(·), i.e., they may be written as(60)

where *d *is the number of knots used in the spline expansion (*d *may be obtained by selecting the value that minimizes the Generalized Cross Validation). *mean*(*H*) and *var*(*H*) (or *mean*(*S*\{*H*}) and *var*(*S*\{*H*})) are vectors containing the mean and variance values of each row of *H *(or *S*\{*H*}), respectively. In this step, the smoothed curves  and  represent the estimated variance for each probe. Notice that the smoothed curve in housekeeping genes and negative controls  represents the estimated measurement error for each gene expression level. Moreover, the smoothed curve in the remaining set of probes  represents the total variance (intrinsic biological variance + measurement error) for each gene expression level;

3. Divide the smoothed curve  (obtained in step 2) by the other smoothed curve . Notice that this ratio () is the estimation of measurement error in percentage of the total variance for each probe. With this percentage, it is possible to estimate the variance of the measurement error for each probe.

### Simulations

In order to evaluate the behavior of both, standard and proposed methods, we have conducted two simulations in small, moderate and large samples sizes (50, 100, 200 and 400). Computations were performed on the R software (a free software environment for statistical computing and graphics) [34]. For each group of simulation, 10,000 Monte Carlo samples were generated. Simulation I is for independent data and Simulation II for time series data.

#### Simulation I - independent data

In order to evaluate the performance of both, usual and corrected OLS methods, a controlled structure was defined. Let *x *and *y *be gene expression values where one is interested in examining if a certain gene *x*_*i *_(*i *= 1,..., *p*; *p *= 9) is linearly correlated to gene *y *(*q *= 1) partialized by other genes. This situation can be represented by the following structure

where

The observed variables *X*_*i *_(*i *= 1,...,9) and *Y *are defined by

where *x*_*i *_~ *N*(0, 1), *ε *~ *N *(0, **Σ**_*ε*_) is the intrinsic biological random variation and ϵ_1 _~ *N*(0, ) independent of ϵ_2 _~ *N*(0, ) are the measurement errors, with  =  varying from 0 to 0.8. The standard deviation **Σ**_*ε *_is defined by

In order to become the simulation more realistic (since actual biological gene expression signals are generally quite correlated), notice that **Σ**_*ε *_is not a diagonal matrix, i.e., there are little correlations between the predictors. The sample's size varied from 50 to 400.

#### Simulation II - time series data

In the time series case, the data has some peculiarities which are not present in the independent data. Time series data are known to be autocorrelated (past values associated with future values) and also contemporaneously correlated (contemporaneous correlation between time series). Considering these characteristics, a similar structure described in the previous section was designed. Let *X*_*t *_and *Y*_*t *_being gene expression time series data and one is interested in verifying if certain gene *x*_*i*, *t *_(*i *= 1,..., *p*; *p *= 9) Granger causes gene *y*_*t *_(*q *= 1). This problem can be modeled by a VAR process of order one as described below:

where

and

The observed variables *X*_*t *_and *Y*_*t *_are defined by

where *ε *~ *N*(0, **Σ**_*ε*_) is the intrinsic biological random variation and *ϵ*_1 _~ *N*(0, ) independent of ϵ_2 _~ *N*(0, ) are the measurement errors, where  varies from 0 to 0.8. The standard deviation **Σ**_*ε *_is defined by

The time series length varied from 50 to 400.

Notice that *β*_0 _is the autoregressive coefficient and all time series *X*_*i *_for *i *= 1,...,9 are autocorrelated and also contemporaneously correlated (**Σ**_*ε *_is not a diagonal matrix).

### Actual biological data

The standard and proposed OLS methods were applied to lung cancer gene expression data collected by [35]. This dataset is composed of 400 microarrays, each of which constructed using a different cDNA obtained from a different patient. Standard and corrected VAR approaches were applied to mouse liver time series data collected by [36]. This data is composed by 48 time points distributed at intervals of 1 hour.

## Authors' contributions

AF has made substantial contributions to the conception, design and implementation of the study, and has also been responsible for drafting the manuscript. AGP has made substantial contributions to the development of the methods. JRS has made contributions to data analysis. SM has discussed the results and critically revised the manuscript. All authors read and approved the final version of the manuscript.

## Appendix

### Proof of the asymptotic variance of *β *- equation (29)

Here, we proof equation (29), i.e., the asymptotic variance of *β *in the multivariate case with no correlated errors.

Consider the following model:(61)

where **X**_*i *_and **Y**_*i *_are the observed vectors with dimensions *p *× 1 and *q *× 1, respectively, *α *is the model intercept (*q *× 1), *β *is a (*q *× *p*) matrix of slope parameters, *ε *_*i *_is a white noise vector with mean zero and covariance matrix **Σ**_*ε*_. The joint distribution of ϵ_1*i*_, ϵ_2*i*_, *ε*_*i *_and **x**_*i *_is given by(62)

In this section, we investigate the asymptotic distribution of

where

and

The proof idea, similar to presented in [23], has two main steps. The first step consists in showing that vec() - vec(*β*^⊤^) can be written as linear combinations of a vectorial mean. In the second one, we must demonstrate that this vectorial mean has an asymptotic normal distribution. Therefore, we need some auxiliar results for proving the asymptotic result, which are exposed in two propositions below.

**Proposition 1 **Under the model (61) under (62) the proposed estimator  has the following relationship(63)

where

with **W**_*i *_= (*ε*_*i *_+ ϵ_2*i *_- *β*ϵ_1*i*_) ⊗ (**x**_*i *_- *μ*_*x *_+ ϵ_1*i*_) - **Ψ**, **Ψ **= (**I**_*q *_⊗ )vec(*β*^⊤^) and *b*_*n *_=  means that *nb*_*n *_is limited in probability when *n *diverges. It implies that, _*prob*_(*n*^-1^) goes to zero when *n *increases.

**Proof: **Define *β*_*k *_as the coefficients associated with the *k*^*th *^element of the vector **y**_*i*_, that is

Thus, we have that vec(*β*^⊤^) = ()^⊤ ^and its estimator can be written as , where  and  for *k *= 1,..., *q*. Moreover, the model (61) may be rewritten in terms of the observed variables as(64)

and for the *k*^*th *^element of **Y**_*i *_we have(65)

where ϵ_2*i *_= (ϵ_2,1*i*_,...,ϵ_2, *qi*_)^⊤^.

Then, it follows that

where . Thus, denoting  we have that

with . As a result, we have

where  and **W**_*ki *_= (**x**_*i *_- *μ*_*x *_+ ϵ_1*i*_)ϑ_*ki *_- **Ψ**_*k*_. Hence, it follows that(66)

where

with **W**_*i *_= (*ε*_*i *_+ ϵ_2*i *_- *β*ϵ_1*i*_) ⊗ (**x**_*i *_- *μ*_*x *_+ ϵ_1*i*_) - **Ψ **and **Ψ **= (**I**_*q*_ ⊗ ) vec(*β*^⊤^).

**Proposition 2 **Under all conditions stated in this paper, the mean  of Proposition has an asymptotic distribution given by

where  means "converge in distribution to",

and 

**Proof: **Notice that the expectation of **W**_*i *_is equal to zero for all *i*. Then, defining , where *x*_*i *_= *δ*^⊤^**W**_*i *_we have that E(*x*_*i*_) = 0, Var(*x*_*i*_) = *δ*^⊤^E(**W**_*i *_)*δ *and

with **F**_*i *_= (*ε*_*i *_+ ϵ_2*i *_- *β*ϵ_1*i*_) (*ε*_*i *_+ ϵ_2*i *_- *β*ϵ_1*i*_)^⊤^. Thus, using the fact that the random quantities have independent normal distributions and we have that

That is, *x*_1_..., *x*_*n *_is an iid sequence and we can use the central limit theory, which says that

where *V *= *δ*^⊤ ^**T***δ *with . As  is asymptotically normally distributed for all *δ *≠ **0**_*r *_then, by the Cramer-Wold device [37], we have that

Then, by the Propositions (1) and (2), we have that

where .

### Dahlberg's error

Consider the following model:(67)

where *Z*_*ij *_is the measure obtained in one experiment (microarray), *i *is the sample index *i *= 1,..., *m*, *m *is the number of spost in the microarray, *j *is the replicate number (*j *= 1, 2 in the case of duplicates), *μ*_*i *_is the unknown true value of the measure and ϵ_*ij *_is the error of measure.

Then, assume that *E*(ϵ_*ij*_) = 0 and *Var*(ϵ_*ij*_) = . Thus, one quantification of the quality of measure is the standard deviation of ϵ_*ij*_, i.e., *δ*_ϵ_. Notice that the lower is the standard deviation of the error of measure (*δ*_ϵ_), the lower is the measurement error.

Consider(68)

Therefore(69)

Assuming that there is no bias (systematic error), one intuitive estimator for  is(70)

The quantity  is exactly the Dahlberg's formula proposed in [30].
